# P2X7 Cell Death Receptor Activation and Mitochondrial Impairment in Oxaliplatin-Induced Apoptosis and Neuronal Injury: Cellular Mechanisms and *In Vivo* Approach

**DOI:** 10.1371/journal.pone.0066830

**Published:** 2013-06-27

**Authors:** France Massicot, Guillaume Hache, Ludivine David, Dominique Chen, Charlotte Leuxe, Laure Garnier-Legrand, Patrice Rat, Olivier Laprévote, François Coudoré

**Affiliations:** 1 Laboratoire de Chimie-Toxicologie Analytique et Cellulaire, EA4463, Faculté des Sciences Pharmaceutiques et Biologiques, Université Paris Descartes, PRES Sorbonne Paris Cité, Paris, France; 2 Laboratoire de NeuroPharmacologie, EA3544, Faculté de Pharmacie, Université Paris Sud 11, PRES Paris-Saclay, Chatenay-Malabry, France; National Institutes of Health, United States of America

## Abstract

Limited information is available regarding the cellular mechanisms of oxaliplatin-induced painful neuropathy during exposure of patients to this drug. We therefore determined oxidative stress in cultured cells and evaluated its occurrence in C57BL/6 mice. Using both cultured neuroblastoma (SH-SY5Y) and macrophage (RAW 264.7) cell lines and also brain tissues of oxaliplatin-treated mice, we investigated whether oxaliplatin (OXA) induces oxidative stress and apoptosis. Cultured cells were treated with 2–200 µM OXA for 24 h. The effects of pharmacological inhibitors of oxidative stress or inflammation (N-acetyl cysteine, ibuprofen, acetaminophen) were also tested. Inhibitors were added 30 min before OXA treatment and then in combination with OXA for 24 h. In SH-SY5Y cells, OXA caused a significant dose-dependent decrease in viability, a large increase in ROS and NO production, lipid peroxidation and mitochondrial impairment as assessed by a drop in mitochondrial membrane potential, which are deleterious for the cell. An increase in levels of negatively charged phospholipids such as cardiolipin but also phosphatidylserine and phosphatidylinositol, was also observed. Additionally, OXA caused concentration-dependent P2X7 receptor activation, increased chromatin condensation and caspase-3 activation associated with TNF-α and IL-6 release. The majority of these toxic effects were equally observed in Raw 264.7 which also presented high levels of PGE2. Pretreatment of SH-SY5Y cells with pharmacological inhibitors significantly reduced or blocked all the neurotoxic OXA effects. In OXA-treated mice (28 mg/kg cumulated dose) significant cold hyperalgesia and oxidative stress in the tested brain areas were shown. Our study suggests that targeting P2X7 receptor activation and mitochondrial impairment might be a potential therapeutic strategy against OXA-induced neuropathic pain.

## Introduction

Platinum derivatives are among the most commonly used anticancer drugs. One of them, oxaliplatine (OXA), produces fewer DNA adducts than cisplatin at equimolar concentrations but also causes higher cytoxicity [1], [2]. Indeed, the therapeutic use of OXA in metastatic colorectal cancer is often limited by neuropathies whose severity of symptoms depends on the patient and on the cumulative dose. Acute neurotoxicity may appear as soon as after the first injection and symptoms (cold-induced paresthesia and cramps) disappear within a week following injection, whereas chronic painful signs resulting from cumulative OXA doses were only reversed several months after treatment discontinuation [3].

The causes of OXA-induced neurotoxicity are not yet well known and there is no indication that a common mechanism induces both the acute and chronic toxicity. In the dorsal root ganglion (DRG) neurons but not in hippocampal neurons, a Na^+^ channelopathy [4], [5] has been proposed as the mechanism underlying acute neuropathy which is mediated through changes in transient rather than persistent Na^+^ conductance [4]. The sodium channel isoform Na_v_1.6 seems to play a central role in mediating acute cooling-exacerbated symptoms following OXA. These enhanced and persistent sodium currents may provide a general mechanistic basis for cold-aggravated symptoms of neuropathy [5]. OXA could also promote hyperexcitability by remodelling ion channel expression in cold-sensing nociceptors [6]. Chronic OXA neurotoxicity is linked to its accumulation in the DRG and to the decrease in conduction velocity of sensory nerves. DRG channelopathy can also be involved in the cortical area where a down-regulation of potassium channels could result from a down-regulation of several genes. Five genes coding for potassium channels were found and a down-regulation of voltage-gated potassium (Kv2.2) channel gene expression was demonstrated. This OXA-induced reduction of Kv2.2mRNA may lead to a reduction in K+ currents and contribute to hyperexcitability and spontaneous ectopic discharges in the somatosensory cortex [7]. Sensory neurons express several members of the transient receptor potential (TRP) family of ion channels including TRP ankyrin 1 (TRPA1) channel which contributes to cold hypersensitivity, via generation of oxidative stress [8]. Channel activation is most likely caused by glutathione-sensitive molecules, including reactive oxygen species and their byproducts, which are generated after tissue exposure to platinum-based drugs from cells surrounding nociceptive nerve terminals. Thus, pain can be an early manifestation of a process that may ultimately lead to neuronal cell death.

At the cellular level, the chemotherapy interferes with DNA replication and metabolic function of the neurons [9]. The amount of DNA cross-links in DRG neurons is significantly correlated with the degree of neurotoxicity [10]. OXA treatment induced a region-specific up regulation of protein kinase C within thalamus, and periaqueductal grey matter, two sites which are implicated in nociception [11]. In DRG neurons, a loss of phosphorylated neurofilament causes nerve injury and produces sensory/motor deficits [12]. It was also suggested that OXA acts on isolectin B4 (IB4)-positive nociceptors to induce oxidative stress-dependent acute peripheral sensory neuropathy [13]. However the mechanisms of the neurotoxic painful effect have been poorly investigated even if an activation of apoptotic pathways and involvement of oxidative stress were mentioned [14]–[16]. Chronic pain characterized by hyperalgesia or allodynia results from a neurochemical and phenotype sensitization of peripheral and central sensory nerves [17], [18] implicating various receptors. In particular, Mihara et al, 2011 [19] have recently shown that spinal NR2B-containing N-methyl-D-aspartate (NMDA) receptors contribute to OXA-induced mechanical allodynia in rats. Multiple purinoreceptor subtypes such as P2X4, P2X7 and P2Y12 are also involved in pain pathways both as initiators or modulators (Burnstock, 2009) [20]. P2X7 receptors are now a new target for inflammatory neuropathic pain (Donelly-Roberts et al., 2008) [21]. These receptors are members of the family of ionotropic ATP-gated receptors. They express their activity through the nervous system but are readily detectable in cells of hemopoietic lineage including macrophages and microglia and mediate the influx of Ca^2+^ and Na+ as well as the release of pro-inflammatory cytokines [22]–[24]. P2X7R activation may thus exert a strong role in degenerative inflammatory diseases as well as in inflammatory and neuropathic pain [23]–[27]. In this study, we have thus tested P2X7R involvement in our *in vitro* and *in vivo* models.

The prevention of OXA-induced neuropathy is of great need for limiting such debilitating side-effects. There is no clear consensus regarding the treatment of the painful symptoms and several substances have been clinically tested [18], [28], but they have so far proven unsuccessful. Although the mechanism underlying the side effects induced by platinum anticancer drugs is not clearly understood, it could be attributed to the combination of several factors such as the generation of reactive oxygen species (ROS) which could interfere with the antioxidant defence system resulting in oxidative damage in different tissues [16], [29], and reaction with thiols in protein and glutathione, causing cell dysfunction. This implication of oxidative stress in cisplatin side effects is clearly documented in the literature whereas it was only recently evoked with oxaliplatin [13], [16]. Thus, thiols such as N-acetylcysteine (NAC) are increasingly used in clinical trials of platinum chemotherapy [30], [31] and it has been demonstrated that the efficacy of chemotherapy was not affected by chemoprotection [31], [32]. NAC also caused analgesia in a model of chronic inflammatory pain [33]. These results prompted us to investigate the effects of NAC in oxaliplatin toxicity. Non-steroidal anti-inflammatory drugs (NSAIDs) are primarily used for the treatment of acute or chronic conditions with pain and inflammation. Evidence from a wide range of sources suggested that chronic administration of NSAIDs reduced the risk of cancer incidences [34] and NSAIDs have emerged as significant chemopreventive agents against several cancers [35]. Therefore, we have looked for preventive effects of Ibuprofen, a well-tolerated pain reliever with anti-inflammatory activity. A second pain reliever, acetaminophen, has also been studied because it is often considered as a first-line approach to pain management [36] although there is a risk of hepatoxicity at high doses.

The understanding of the pathophysiological mechanisms of OXA-induced neurotoxicity was thus our major goal for a most effective treatment. The neuroblastoma SH-SY5Y cell line was used as a neuronal model and we have particularly focused on markers of oxidative stress, mitochondrial activity and apoptosis of cells. Our results were compared with those obtained in OXA-treated mice. It is suggested that macrophage activation could be involved in neuropathic pain mechanisms [37], [38]. Therefore, OXA effects were evaluated on a macrophage model (Raw 264.7 cell line). The potential use of putative neuro-protectants (as anti-inflammatory and anti-oxidative drugs) in the prevention of the OXA-induced neuropathy is discussed.

## Materials and Methods

### Materials

Materials for cell culture were provided by Corning. Tissue culture medium was from Eurobio (Montpellier, France). All chemicals were obtained from Sigma-Aldrich (Saint-Quentin Fallavier, France), except fluorescent probes which were purchased from Invitrogen (Villebon-sur-Yvette, France) or Interchim (Montluçon, France). Il-1β, Il-6 and TNF-α detection kits were from Raybiotech (USA).

### Animal treatment

Experiments were performed on 7-8-week-old adult male C57BL/6 mice (Janvier, Le Genest Saint Isle, France). Animals were given free access to standard food and water, with a 12 h light-dark cycle at a temperature of 22+/−2°C. All experiments were carried out in the central animal facility (D75-0602) of the Faculty of Pharmaceutical and Biological Sciences, Paris Descartes University in strict accordance with the European Community Council Directive (86/609/EEC) of November 24, 1986. The protocol was approved by the local Ethical Committee on Animal Research (CEEA34), Paris Descartes University, PRES “pôle de recherche et d'enseignement supérieur” Sorbonne Paris Cité. All efforts were made to minimize suffering. To determine the OXA-induced painful neuropathy, mice were repeatedly injected i.p. with 7 mg/kg OXA (n = 10) at days 1, 2, 5 and 6 (28 mg/kg cumulated dose). Behavioral tests and care of animals were conducted in accordance with the guidelines of the International Association for the Study of Pain. Control animals (n = 8) received equal volumes of saline. All animals were euthanized by cervical dislocation under light anaesthesia. Brains were immediately removed, washed twice with PBS buffer and dissected in order to isolate different areas such as frontal cortex, striatum, and hippocampus by direct observation. All anatomical structures were mechanically dissociated and homogenised in ten volumes of ice-cold PBS buffer with a Teflon-glass homogenizer. After centrifugation, the suspended mitochondrial pellet (75-90 µg proteins) was incubated with adequate probes for assessment of the markers of inflammation/oxidative response or stored at −80°C until cytokine analysis.

### Neuronal and macrophage cell culture

The human SH-SY5Y cell line (94030304, ECACC) and RAW 264.7 macrophages (8503803, ECACC) were obtained from Sigma-Aldrich (Saint-Quentin Fallavier, France) and respectively grown in Dulbecco's MEM (DMEM, Life Technologies-Invitrogen, Saint Aubin, France) or in RPMI (Life Technologies-Invitrogen, Saint Aubin, France), each supplemented with 10% heat inactivated foetal bovine serum (FBS) (Eurobio, Montpellier, France), 1% L-glutamine and 1% penicillin/streptomycin (Life Technologies-Invitrogen, Saint Aubin, France) in a 5% CO_2_-95% O_2_ atmosphere at 37°. When confluent, the cells were seeded into 96-multiwell plates (NUNC) at a density of 2×10^5^ cells/mL (100 µl per well).

### Cell treatments

Experiments were carried out 24 h after cells were seeded. OXA was dissolved in methanol to obtain a 20 mM stock solution, which was diluted appropriately at the time of use. Cells were exposed for 24 h to various OXA concentrations 2 µM to 200 µM, a range that includes the therapeutic level in humans [39]. In some experiments only one selected concentration of OXA was used for testing the effects of putative protective drugs. Each experiment was repeated at least three times. The effects of specific pharmacological inhibitors of oxidative stress (N-acetyl cysteine or NAC at 1 mM) and inflammation or pain (ibuprofen or IBU at 1 µM; acetaminophen (N-Acetyl-para-aminophenol) or AAP at 50 µM) were also tested. Inhibitors were added 30 min before OXA treatment and then in combination with OXA for 24 h. NAC, IBU, or AAP tested concentrations were based on literature data, respectively [40]–[44]. Stock solutions of all drugs were prepared in PBS and diluted in 2.5% FBS-containing medium. All assays were run in cell lines in triplicate, and each experiment was repeated three to five times.

### Biochemical analysis

All tests were performed either on SH-SY5Y and RAW 264.7 cells or on tissue homogenates using adapted protocols. The present *in vitro* study was performed directly on living adherent cells without extraction to evaluate the most liable markers concerning cell viability, oxidative stress, apoptosis, using microtitration fluorometric assays (MIFALC tests) as previously described [45]–[48].

### Cell viability

Cell viability was tested through either the intracellular redox status using the Alamar Blue® test, membrane integrity using the neutral red test and mitochondrial metabolism using the 3-[4,5-dimethylthiazol-2-yl]-2,5-diphenyl tetrazolium bromide (MTT) test. The Alamar blue assay uses a visible blue fluorogen probe, resazurin (Sigma-Aldrich, Saint-Quentin Fallavier, France) which is reduced to a red fluorescent compound (resorufin) by cellular redox enzymes [47], [49]. Alamar blue (20 µL) was diluted in culture medium supplemented with 2.5% foetal bovine serum (200 µL). After incubation with the test solutions, the microplate was then incubated with the dye solution during 6 h at 37°C. The Alamar blue fluorescence was then measured at λexc  = 535 nm and λem  = 600 nm using microplate fluorometric detector (Safire; Tecan, Lyon, France). Neutral red was used at a 50 µg/ml concentration [50] and as previously described [46]. Two hundred microliters per well of neutral red solution were added to living cells, and the microplate was incubated for 3 h at 37°C in moist atmosphere with 5% CO_2_. The cells were washed in PBS and the dye was extracted from the intact and viable cells with a solution of acetic acid–ethanol. The plate was agitated on a microplate shaker for 20 min, and then fluorescence was measured using our microplate fluorometric detector. The neutral red fluorescence was then measured at λexc  = 535 nm and λem  = 600 nm. In another set of experiments, 3-[4,5-dimethylthiazol-2-yl]-2,5-diphenyl tetrazolium bromide (MTT; Sigma-Aldrich, Saint-Quentin Fallavier, France) was added to the cell culture medium at 0.5 mg/mL 24 h after OXA addition. Cells were incubated 3 h at 37°C. The medium was then removed, and DMSO was added to dissolve the formazan crystals. The absorbance of the resulting solution was spectrophotometrically measured at 570 nm. The value was directly proportional to the number of the viable cells and activity of mitochondrial metabolism.

### Oxidative stress

#### Reactive oxygen species (ROS) production: DCF-DA test

Free radical generation and ROS, mainly hydrogen peroxide (H_2_O_2_) production, were detected with the 2′,7′-dichloro fluorescein diacetate (DCFH2-DA) probe, (Interchim, Montluçon, France) added for 20 min to living cells after incubation with the tested solutions. The non-fluorescent polar derivative (H2DCF) is rapidly oxidized to give the highly green fluorescent 2′,7′-dichlorofluorescein diacetate, in the presence of intracellular reactive oxygen species, mainly H_2_O_2_. The fluorescent signal (λexc  = 485 nm; λem  = 535 nm) is proportional to ROS production.

#### Superoxide anion production: Dihydroethidium test

Superoxide anion (O2°^−^) was detected using the dihydroethidium probe (Interchim, Montluçon, France). A 1.58 µg/ml solution was prepared and cells were pre-incubated for 20 min. Following its incorporation into cells, dihydroethidium was oxidized to the fluorescent ethidium cation by O2°^−^, allowing the cation to bind to nuclear DNA with an extensive fluorescent enhancement. Fluorescence detection (λexc  = 485 nm; λem  = 535 nm) is proportional to O2°^−^production.

#### Nitrite contents

After incubation with the tested solutions, culture media were collected and nitrite concentration was determined by a spectrophotometric method based on the Griess reaction. Briefly, 100 µL of culture medium or sodium nitrite (Merck, Fontenay-sous-Bois, France) standard dilutions were mixed with 100 µL Griess reagent containing equal volumes of 1% sulphanilamide (Sigma) in 5% phosphoric acid (Merck) and 0.1% N-(1-naphtyl) ethylene diamine solution (Merck, Fontenay-sous-Bois, France) and incubated for 10 min at 37°C. The absorbance was measured at 550 nm.

#### Lipid peroxidation (LPO): Malondialdehyde (MDA) measurement

The content of MDA, an indicator of LPO, was determined using the thiobarbituric acid (TBA) method and performed according to the procedure described by Yagi [51]. Briefly, the 24-h supernatant of the OXA-treated cells was collected and used for measuring absorbance at 532 nm. 1, 1, 3, 3′-tetraethoxypropane was used as an external standard. The results are expressed as nanomoles of MDA formed per mg of protein. The protein content of cell lysates was determined using bicinchoninic acid method with bovine serum albumin as standard (Sigma-Aldrich, Saint-Quentin Fallavier, France).

### Apoptosis

#### Mitochondrial potential: JC-1 test

JC-1 probe (Life Technologies-Invitrogen, Saint Aubin, France), a cationic and lipophilic dual fluorescence dye, allows the determination of variations of mitochondrial transmembrane potential (Δφ_m_). The fluorescence emission of JC-1 depends on the probe state. The probe forms aggregates when mitochondrial activity is high (hyperpolarisation: red fluorescence) and monomers when mitochondrial activity is low (green fluorescence). The dye solution (6.5 µg/mL in phosphate buffer saline PBS) was added to living adherent cells after 24-h incubation time with the tested solution. The microplate was incubated at 37°C during 15 min and the read at λexc  = 485 nm, λem  = 520 nm. The accumulation of dye aggregates is indicated by a fluorescence shift from green (emission 530 nm) to red (emission 590 nm). The Δφ_m_ value is determined by the ratio of green to red fluorescence data.

#### Mitochondrial negatively charged phospholipids: Nonyl Acridine Orange test

Mitochondrial negatively charged phospholipid levels, mainly cardiolipin but also phosphatidylserine and phosphatidylinositol were evaluated using the nonylacridine orange fluorescence probe (Life Technologies-Invitrogen, Saint Aubin, France) which stains all phospholipids present on either the outer or the internal leaflet of the inner mitochondrial membrane. The microplate was incubated with the dye solution (10 µM in culture medium) for 30 min. at 37°C. After 1 h recovery period, cells were washed in PBS. The dye was extracted from viable cells with a solution of acetic acid-ethanol. After agitation on a microplate shaker for 30 min, the microplate was read at λexc  = 490 nm and λem  = 530 nm.

#### Cell death P2X7 receptor activation: YOPRO-1 test

YOPRO-1 (Invitrogen) is a DNA probe usually used to discriminate cells dying by apoptosis versus necrosis with both flow cytometry [52] and fluorescence microscopy [53], [54]. Yopro-1 enters apoptotic cells after P2X7 receptor activation-induced pore formation. Experiments were conducted using microplate cytofluorometry which allows the use of fluorescent probes directly on living cells and detect the fluorescent signal directly in the microplate in less than 1 min (for a 96 well microplate) [54]–[56] After incubation with the tested solutions, a 2 µM YOPRO-1 solution in phosphate buffer saline (PBS) was distributed in the wells (200 µL), and the microplate was placed at room temperature in the dark. After 10 min, the fluorescence signal was detected at λexc  = 491 nm and λem  = 509 nm. The fluorescent signal was viewed with a microscope. In this assay, cells were also pre-incubated for 30 min with 10 µM Brillant Blue G (BBG from Bio-Rad) (Richmond, CA), a selective P2X7 receptor antagonist [57]–[59], before exposition to OXA concentrations.

#### Chromatin condensation assay: Hoechst 33342 probe

Hoechst 33342 (Invitrogen) is a non-cytotoxic DNA-binding dye that permits the determination of the total chromatin quantity variation and the degree of chromatin condensation [60]. It preferentially binds to triplet adenine and thymidine base pairs. This probe was used on cells at a final concentration 10 µg/ml. Cells were then examined under a fluorescence microscope as validated by Du et al., [61], Mc Caffrey et al., [62], and Palmer et al. [63] and after 30 min, the fluorescent signal was detected at λexc  = 360 nm and λem  = 450 nm according to Rat et al. [45] and Kasseckert et al. [64]. Propidium iodide (from Sigma-Aldrich, Saint-Quentin Fallavier, France) at 0.5 mg/ml was added to the Hoechst solution to control necrotic cells (this DNA probe reacts by intercalation and does not allow the Hoechst dye fixation by necrotic cells), as previously validated [65].

#### Quantification of caspase-3 activity by colorimetric assay

The apoTarget^TM^ Caspase-3 Protease assay (Life Technologies-Invitrogen, Saint Aubin, France) was used for the in vitro determination of caspase-3 proteolytic activity in selected brain areas of OXA-treated mice or in lysates of SH-SY5Y treated either with OXA alone or with OXA plus either acetaminophen (AAP), ibuprofen (IBU), or N-acetyl cysteine (NAC), as described by the manufacturer's instructions. Briefly, cytosol extracts (≈ 200 µg prot) were incubated at 37°C with a specific peptide substrate for 2 hours. Upon cleavage of the substrate by caspase-3, free chromophore, p-nitroanilide light absorbance was measured at 400 nm. Fold-increase in Caspase-3 activity was determined by direct comparison to the level of the control.

### Inflammatory response

#### Quantification of interleukin (IL)-1β (IL-1β), IL-6 and tumor necrosis factor (TNF)-α release by ELISA

After pre-treatment with AAP, IBU, or NAC, the neuronal cell line SH SY5Y was treated with OXA for 24 h in 24-well plates. The supernatant was collected, centrifuged and stored immediately at −80°C until analysis. Tumor necrosis factor-α (TNF-α), interleukine-1β (IL-1β) and interleukine-6 (IL-6) enzyme-linked immunosorbent assay (ELISA) kits (eBioscience, Paris, France) were used to measure these cytokines (pg/mL) according to the manufacturer's protocol. Briefly, plates were coated overnight at 4°C with appropriate antibody. After adding detection antibody, plates were incubated with Avidin-horseradish peroxidase and absorbance was measured at 450 nm.

#### Quantification of prostaglandin E2 (PGE2) release by enzyme immunoassay

This assay, based on the competition between PGE2 and a PGE2-acetylcholinesterase (AChE) conjugate for a limited amount of PGE2 monoclonal antibody, was used to determine the release of PGE2 in RAW 264.7. Cells were seeded at 5.10^4^ cells per well in 96-well plates. They were treated either with AAP, IBU or NAC which were added to the culture medium for 30 min before adding OXA (50, or 100 or 200 µM). The treated cells were incubated at 37°C for 24 h. The medium was collected in a microcentrifuge tube and centrifuged at 2800 g for 10 min. The supernatant was decanted and the amount of PGE2 determined using a PGE2 Enzyme-Immuno-Assay kit (Imgenex-CliniSciences, Nanterre, France) using Ellman's reagent. Absorbance was measured at 412 nm. Increase in PGE2 content was determined by direct comparison to the level of the control.

### Behavioural assessment of cold hyperalgesia

The cold plate test was performed to assess nociception to a cold painful stimulus [66]. Mice were placed individually on a cold plate at 2.0±0.3°C, with four 30.5-cm Plexiglas walls (Bioseb, Vitrolles, France). Latency of the first jump (in seconds) was measured as painful response by an experimented observer blinded to drug treatment. The mice were then immediately removed. The cut-off was set at 180 s to avoid tissue damage. The cold plate surface was cleaned and dried prior to testing each animal.

### Data analysis

All data are expressed as the mean ± standard error of mean (S.E.M.). Graphs show fluorescence values expressed as percentage of control. Statistical analysis was performed using one-way ANOVA followed by a Fisher test for multiple comparisons between groups (α risk = 0.05) with Sigma Stat 2.0 (Chicago, Illinois). The *in vitro* assays were repeated three to five times. In every case, a difference was accepted as significant if p<0.05. In the cold plate test, we used the Kaplan-Meier survival analysis due to the lack of normal distribution of the data (measurement of latency with a cut-off). Mantel-Cox log-rank test was used to evaluate differences between experimental groups as described by Rainer et al. [67].

## Results

### Biochemical effects of oxaliplatin on neuronal cells and macrophages

**Figure 1:**
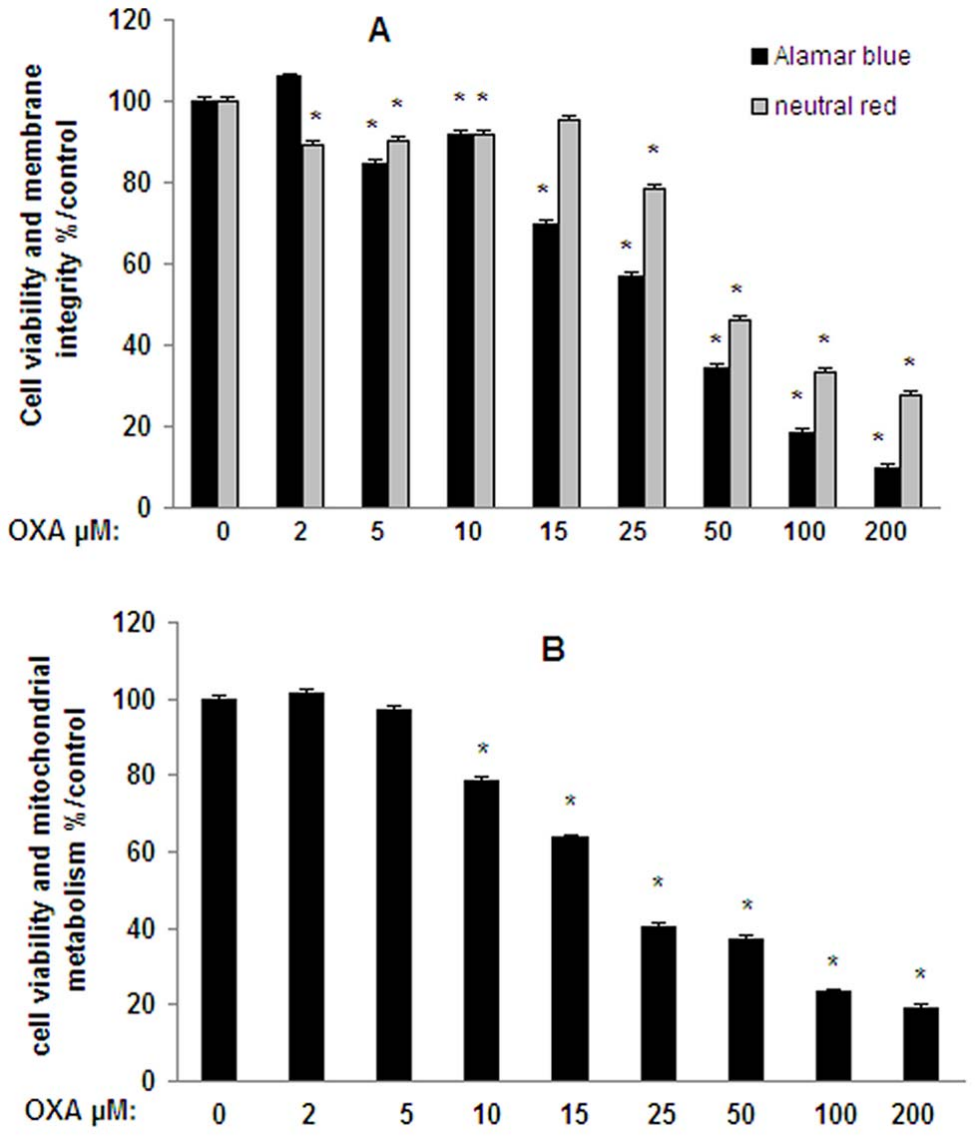
Cell viability and membrane integrity in SH-SY5Y cells exposed to oxaliplatin. Cells (2×10^5^ cells/well) were exposed 24 h to oxaliplatin (OXA) (2–200 µM). Cell viability (redox potential) was evaluated with Alamar blue and membrane integrity was evaluated with neutral red tests (A). Cell viability which is indicative of mitochondrial metabolism was evaluated with MTT test (B). Values are the mean ± S.E.M. expressed as percentage of the control, five different assays per group. *: statistically different (p<0.05) from the mean values in control cells.

In SHSY5Y neuronal cells, a significant decrease in cell viability and cell integrity was found with oxaliplatine (OXA) at all concentrations (up to 70%) for neutral red test (Fig. 1A). The intracellular redox status was evaluated with the Alamar blue test which is also representative of cell viability (Fig. 1A). After a 24-h incubation time, OXA solutions induced a significant dose-dependent decrease (up to 90%) in mitochondrial metabolism. Further investigation was performed with the MTT Test. After 24 h a significant dose-dependent decrease (22% up to 80%) was observed between 10 µM and 200 µM (Fig. 1B). After a 16-h incubation time, cell viability was only decreased by 30% with the higher concentration (200 µM) (not shown). Thus, OXA seems to induce a time-dependent decrease. These results obtained with the MTT test confirm those obtained with the Alamar blue probe.

**Figure 2:**
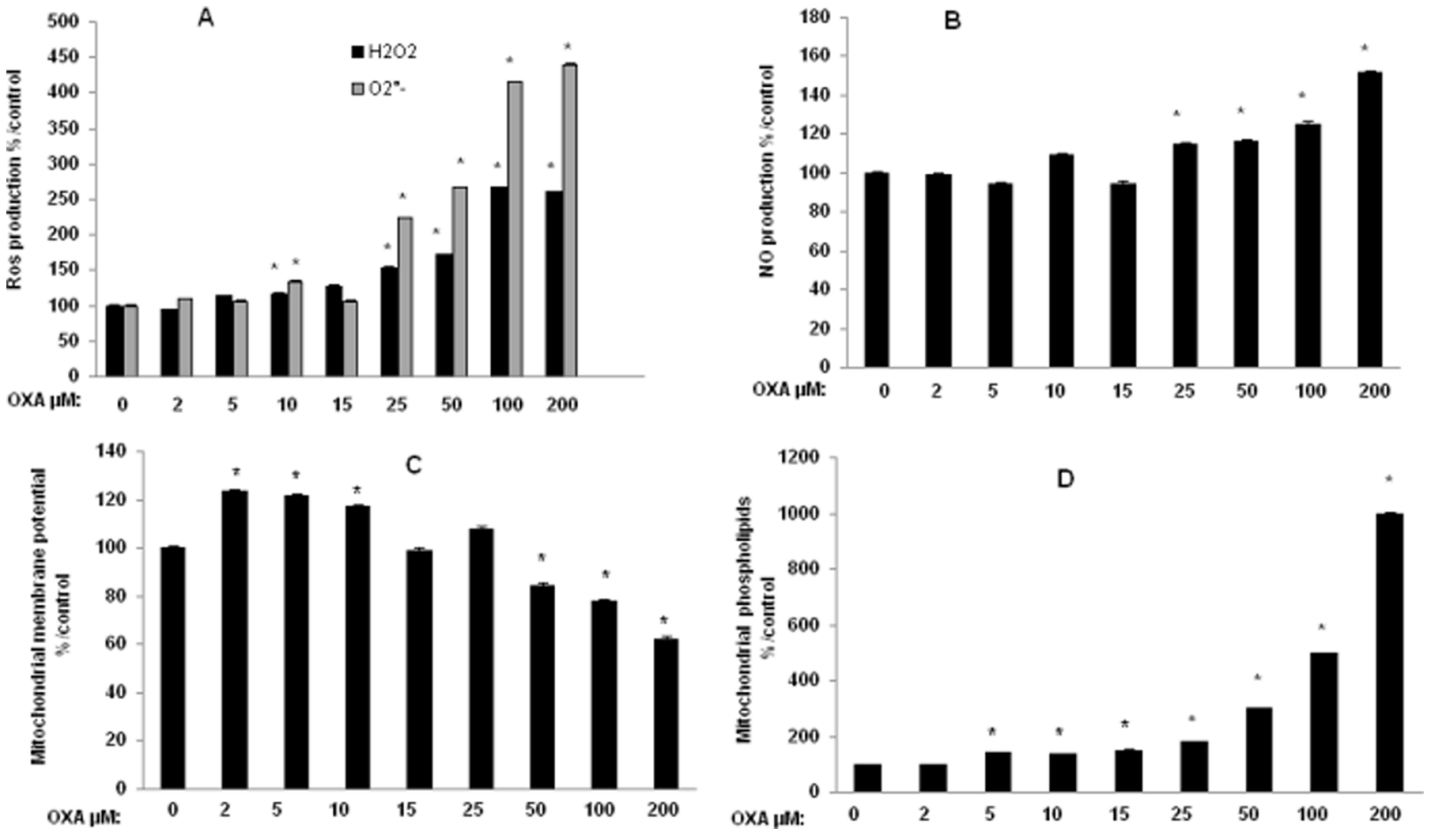
Oxidative stress and mitochondrial activity in SH-SY5Y cells exposed to oxaliplatin. Cells (2×10^5^ cells/well) were exposed 24 h to oxaliplatin (OXA) (2–200 µM). Oxidative stress was evaluated by Reactive Oxygen Species (ROS) production using dihydroethidium and DCF-DA tests (A) and Nitric Oxide (NO) production evaluated as nitrite content (B) by the Griess reaction. Mitochondrial activity was evaluated by determining mitochondrial membrane potential (Δφ_m_) (C) using JC-1 test and mitochondrial levels of negatively charged phospholipids, mainly cardiolipin, using nonyl acridine orange test (D). Values are the mean ± S.E.M. expressed as percentage of the control, five different assays per group. *: statistically different (p<0.05) from the mean values in control cells.

OXA-induced MTT reduction could be due to various processes, including oxidative stress. Therefore, we investigated the effects of OXA on reactive oxygen species (ROS) production in SH-SY5Y cells to determine whether the OXA-induced decrease in mitochondrial redox potential is associated with oxidative stress. A moderate increase in the production of hydrogen peroxide (H_2_O_2_) was detected at the lowest concentration of 10 µM OXA (17% compared with incubation of the vehicle alone) whereas 15 µM up to 200 µM OXA induced a large increase (30% up to 160%) in H_2_O_2_ production (Fig. 2A). OXA also induced a moderate increase at 10 µM (35%) but a significant overproduction of superoxide anions (123% up to 340%) at the higher concentrations (25 µM up to 200 µM) (Fig. 2A). Nitric oxide (NO) production, evaluated as nitrite content, was also significantly increased (15% up to 51%) between 25 and 200 µM (Fig. 2B).

We also wanted to determine whether the OXA-induced oxidative stress caused mitochondrial impairment. Mitochondrial membrane potential and mitochondrial cardiolipin are two important indicators of mitochondrial function. To measure mitochondrial membrane potential, we stained SHSY5Y cells with the fluorescent probe JC-1. OXA led to an increase (≈20%) between 2 and 10 µM and to a decrease in green/red fluorescence intensity ratio (16 up to 38%) showing a loss of mitochondrial membrane potential between 50 µM up to 200 µM OXA (Fig. 2C). Mitochondrial levels of negatively charged phospholipids, mainly cardiolipin, evaluated with nonyl acridine orange probe, were dose-dependently increased between 5 and 200 µM (Fig. 2D). These mitochondrial phospholipids were 5 and 10 times higher at two highest concentrations (100 and 200 µM).

**Figure 3:**
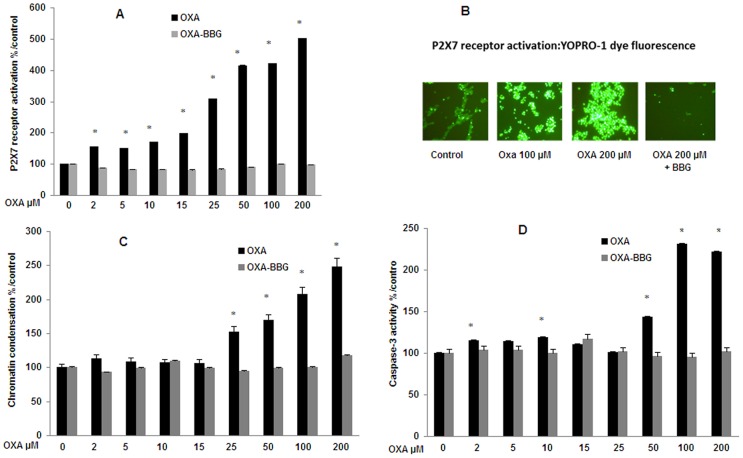
P2X7 receptor activation, chromatin condensation and caspase-3 activity in SH-SY5Y cells exposed to oxaliplatin. Cells were exposed 24 h to oxaliplatin (OXA) (2–200 µM). P2X7 receptor activation (A) was evaluated using YOPRO-1 test. The resulting fluorescence was viewed with a microscope (B). Chromatin condensation (C) was evaluated using Hoechst 33342 test. Caspase-3 proteolytic activity (D) in lysates of cells was evaluated using the apoTarget^TM^ Caspase-3 Protease assay. In these tests, cells were exposed or not to a 30-min pre-treatment with the specific P2X7 receptor antagonist Brilliant Blue G (BBG; 10 µM) prior to OXA for an additional 24 h. Values are the mean ± S.E.M. expressed as percentage of the control, five different assays per group. *: statistically different (p<0.05) from the mean values in control cells.

Increasing OXA concentrations from 2 µM to 200 µM strongly enhanced the YOPRO-1 fluorescence (from 55% up to 400%) indicating a dose-dependent activation of the P2X7 cell death receptor at any tested concentration (Fig. 3A and 3B). A maximal increase (3 up to 5 times) was observed at the highest concentrations (between 25 µM up to 200 µM). This P2X7 activation was totally inhibited by the specific P2X7 receptor antagonist Brilliant Blue G (Fig. 3A and 3B). After 24 h of incubation, the Hoechst 33342 test showed a concentration-dependent increase in fluorescence (52% up to 149%) from 25 µM up to 200 µM, indicating a modification of chromatin condensation (Fig. 3C). Caspase-3 activity was significantly increased by 10% at 2 µM up to 120% at 100 and 200 µM (Fig. 3D). In contrast, increased chromatin condensation and caspase-3 activity were totally inhibited by BBG (fig. 3 C and 3D).

Cytokine release was assessed only at the highest OXA concentration (200 µM). OXA stimulated TNF-α (171%, p<0.0001) and mainly IL-6 release (537%; p<0.001) (Table 1). IL-1β level was slightly increased (57%).

**Table 1:** Cytokines and PGE2 release in oxaliplatin-treated cells after acetaminophen, ibuprofen or N-acetyl cysteine treatment.

Treatment		SH-SY5Y cells		RAW 264.7
	TNF-α pg/ml	IL-6 pg/ml	IL-1β pg/ml	PGE2 pg/ml
Control	7±1	19±7	59±8	100±7
OXA 200 µM	19±2*	121±5*	93±7*	132±6*
OXA+AAP	ND	25±11^$^	70±1^$^	109±5^$^
OXA+IBU	ND	53±12^$^	87±3^$^	111±7^$^
OXA+NAC	ND	25±6$	68±10	109±5^$^

SH-SY5Y cells and RAW 264.7 cells were exposed 24 h to oxaliplatin (OXA) (200 µM) with a 30-min pre-treatment either with acetaminophen (AAP, 50 µM), ibuprofen (IBU, 1 µM) or N-acetyl cysteine (NAC, 1 mM). SH-SY5Y cell supernatant was collected and TNF-α, IL-1β and IL-6 levels (pg/mL) were assessed using ELISA kits according to the manufacturer's instructions. Prostaglandin E2 (PGE2) levels were determined in RAW 264.7 supernatant using a PGE2 Enzyme-Immuno-Assay kit. Values are the mean ± S.E.M. levels in pg/ml, five different assays per group. Significance of differences: OXA alone versus control, *p<0.05; AAP, or IBU, or NAC versus OXA alone: ^$^ p<0.05.

In macrophages, increasing OXA concentrations from 25 µM to 200 µM decreased membrane integrity from 77±13% up to 27±2% and mitochondrial potential metabolism from 77±10% up to 12±2% (Table 2), enhanced NO production from 61% up to 284% (Table 2) but not ROS production (result not shown), and provoked mitochondrial impairment by decreasing mitochondrial membrane potential from 88±6% up to 74±5% from 50 µM up to 200 µM and increasing levels of negatively charged phospholipids from 31% up to 278%. P2X7 receptor activation was increased from 157% up to 330% by OXA (25 µM up to 200 µM) (Table 3) and chromatin condensation was increased from 39% up to 93% with OXA (25 up to 200 µM) (Table 3). Caspase-3 activity was increased between 25 and 200 µM, from 37% up to 428% (Table 3). Prostaglandin E2 (PGE2) release was assessed only at the three highest OXA concentrations 50, 100 and 200 µM, which stimulated PGE2 release from 14% (not shown) up to 32% (200 µM)(Table 1).

**Table 2:** Viability and oxidative stress in RAW 264.7 cells exposed 24 h to oxaliplatin (25–200 µM).

	Viability		Oxidative stress		
	Membrane integrity %	mitochondrial metabolism %	NO %	Δφ_m_ %	m-phospholipids %
Control	100±3	100±5	100±5	100±14	100±4
OXA 25 µM	77±13*	77±10*	161±20*	118±18*	131±4*
OXA 50 µM	32±3*	40±3*	250±5*	88±6	284±7*
OXA 100 µM	37±9*	30±6*	292±1*	94±4	292±2*
OXA 200 µM	27±2*	12±2*	384±9*	74±5*	378±4*

Viability was evaluated both by membrane integrity using neutral red test and mitochondrial metabolism using MTT test. Oxidative stress was evaluated as nitric oxide (NO) production, evaluated as nitrite content by the Griess reaction. Mitochondrial activity was evaluated by determining mitochondrial membrane potential (Δφ_m_) using JC-1 test and mitochondrial levels of negatively levels of charged phospholipids (m-phospholipids), mainly cardiolipin, using nonyl acridine orange test. Values are the mean ± S.E.M. expressed as percentage of the control, six different assays per group. *: statistically different (p<0.05) from the mean values in control cells.

**Table 3:** P2X7R activation, chromatin condensation and caspase-3 activity in RAW 264.7 exposed 24 h to oxaliplatin (25-200µM).

	P2X7R activation	Chromatin condensation	Caspase-3 activity
Control	100±3	100±3	100±11
OXA 25 µM	257±15*	139±17*	137±13*
OXA 50 µM	282±9*	183±15*	405±5*
OXA 100 µM	408±7*	214±21*	428±11*
OXA 200 µM	430±10*	193±6*	528±6*

P2X7 receptor (P2X7R) activation was evaluated using YOPRO-1 test and chromatin condensation using Hoechst 33342 test. The apoTarget^TM^ Caspase-3 Protease assay was used for the *in vitro* determination of caspase-3 proteolytic activity in lysates of RAW 264.7 cells. Values are the mean ± S.E.M. expressed as percentage of the control, six different assays per group. *: statistically different (p<0.05) from the mean values in control cells.

### Biochemical effects of potential protective drugs on neuronal SH-SY5Y cells

N-Acetyl cysteine at 1 mM (NAC), ibuprofen at 1 µM (IBU) and acetaminophen at 50 µM (AAP) were evaluated for their effects on OXA-induced cell injury.

No significant effect of any of the parameters of cytotoxicity was observed when neuronal cells were incubated either with AAP, or IBU or NAC alone compared to control cells (results not shown).

Potential protective effects of AAP, IBU and NAC were assessed by pre-incubating the three drugs for 30 min before adding oxaliplatin (50, 100 and 200 µM) for an additional time of 24 h. Only the highest dose of OXA (200 µM) was used for the determination of the protective effect of the three tested drugs on lipid peroxidation, cytokine and PGE2 release.

**Figure 4:**
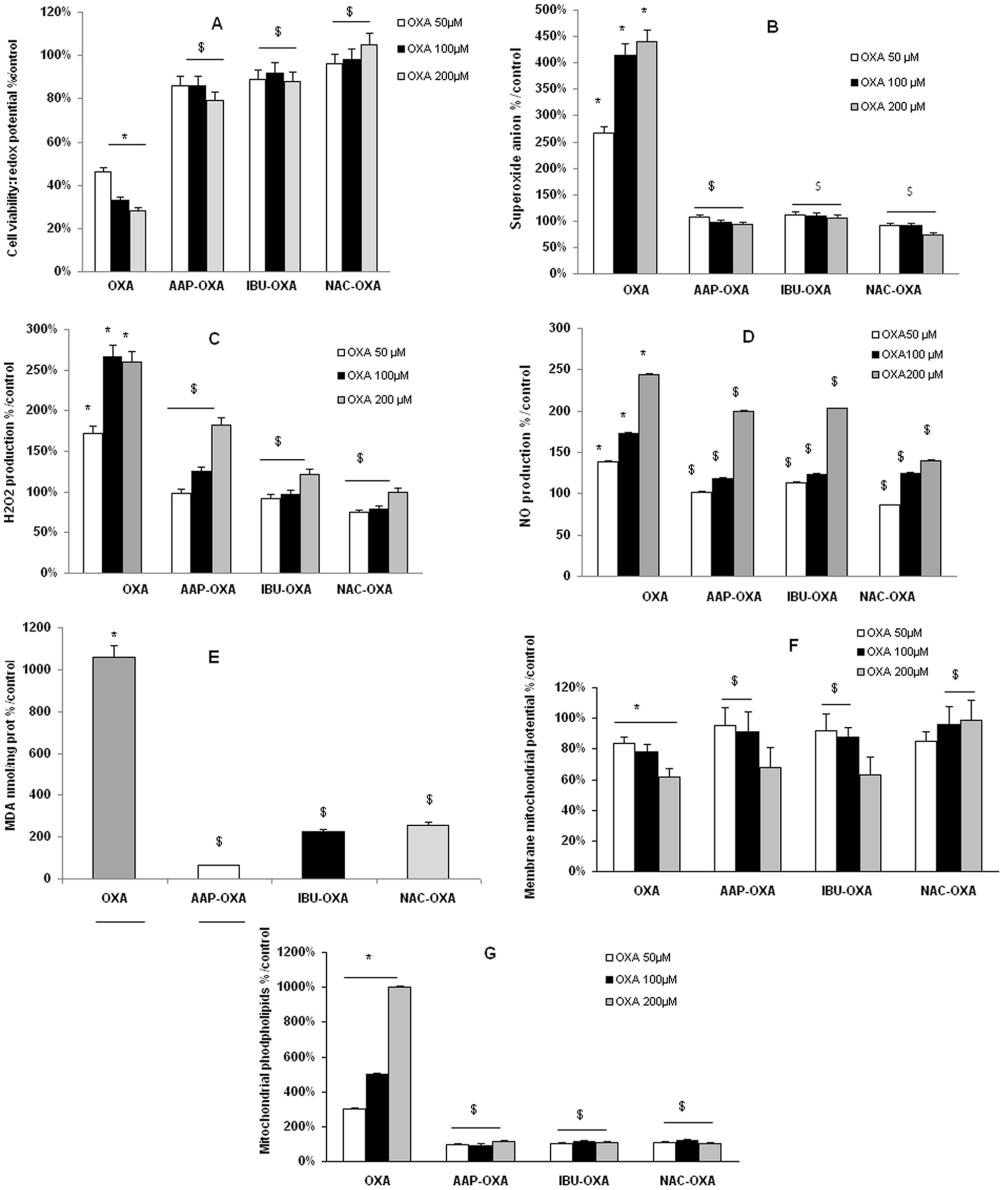
Cell viability and oxidative stress in oxaliplatin-treated SH-SY5Y cells pre-treated by protective drugs. Cells (2×10^5^ cells/well) were exposed for 24 h to oxaliplatin (OXA) (50, 100 or 200 µM) after a 30-min pre-treatment either with acetaminophen (AAP, 50 µM), ibuprofen (IBU, 1 µM) or N-acetyl cysteine (NAC, 1 mM). Cell viability (redox potential) was evaluated with Alamar blue (A). Oxidative stress was evaluated by ROS production using dihydroethidium (B) and DCF-DA (C) tests and nitric oxide (NO) production evaluated as nitrite content (D) by the Griess reaction. Lipid peroxidation (E) was determined using the thiobarbituric acid (TBA) method at 200 µM OXA. Mitochondrial activity was evaluated by determining mitochondrial membrane potential (Δφ_m_) (F) using JC-1 test and mitochondrial levels of negatively charged phospholipids, mainly cardiolipin, using nonyl acridine orange test (G). Values are the mean ± S.E.M. expressed as percentage of the control, five different assays per group. Significance of differences: OXA alone versus control, *p<0.05; AAP, or IBU, or NAC versus OXA alone: $ p<0.05.

Cell viability was significantly restored after cell treatment by either AAP, IBU or NAC whatever the dose of OXA used (Fig. 4A). Moreover, AAP, IBU and NAC have totally inhibited superoxide anion production (Fig. 4B) whereas IBU and NAC were more effective than AAP to induce a nearly total inhibition of H_2_O_2_ production (Fig. 4C). NO production returned nearly to the control levels with the three tested drugs when cells were co-treated with the two lowest OXA concentrations (50 and 100 µM) whereas the decrease was less important when cells were both co-treated with the three tested drugs and with the highest OXA concentration (200 µM) (Fig. 4D). OXA 200 µM induced a 10-fold increase in malondialdehyde (MDA), a marker of lipid peroxidation (p<0.001 *vs* control cells) (Fig. 4E). Lipid peroxidation was strongly decreased by IBU and NAC (_∼_800%) and completely inhibited by AAP. Concerning the mitochondrial membrane potential (ratio green JC-1/red JC-1), a protective effect was observed at the lowest OXA concentrations with AAP and IBU and at the two highest OXA concentration with NAC (Fig. 4F). Mitochondrial levels of negatively charged phospholipids returned to that of the control levels (Fig. 4G).

**Figure 5:**
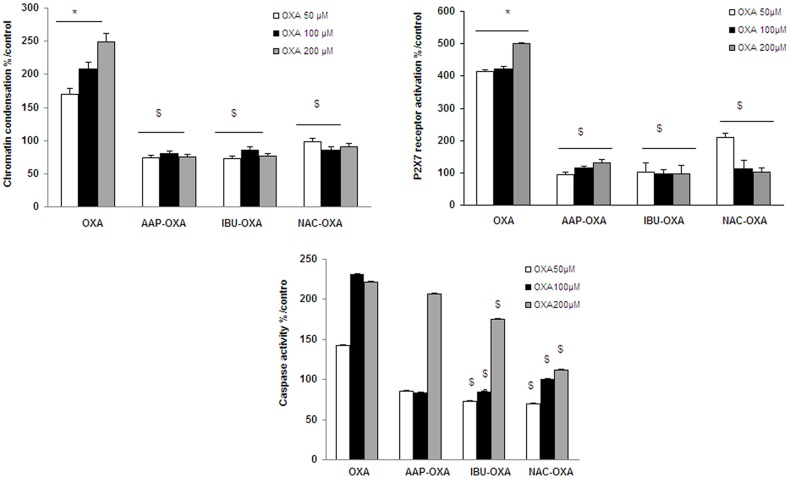
Chromatin condensation, P2X7R activation and caspase-3 activity in oxaliplatin-treated SH-SY5Y cells pre-treated by protective drugs. Cells (2×10^5^ cells/well) were exposed for 24 h to oxaliplatin (OXA) (50, 100 or 200 µM) after a 30-min pre-treatment either with acetaminophen (AAP, 50 µM), ibuprofen (IBU, 1 µM) or N-acetyl cysteine (NAC, 1 mM). Chromatin condensation (A) was evaluated using Hoechst 33342 test and P2X7 receptor (P2X7R) activation (B) using YOPRO-1 test. The apoTarget^TM^ Caspase-3 Protease assay was used for the *in vitro* determination of caspase-3 proteolytic activity (C) in lysates of SH-SY5Y cells as described by the manufacturer's instructions. Values are the mean ± S.E.M. expressed as percentage of the control, five different assays per group. Significance of differences: OXA alone versus control, *p<0.05; AAP, or IBU, or NAC versus OXA alone: $ p<0.05.

Chromatin condensation (Fig. 5A) and P2X7 receptor activation (Fig. 5B) were inhibited with the three tested drugs whatever the OXA concentrations used. Caspase-3 activity was totally inhibited with the two lowest OXA concentrations (50 and 100 µM) whereas the decrease was less important when cells were both co-treated with the three tested drugs and with the highest OXA concentration (200 µM) (Fig. 5C).

Pre-treatment of SH SY5Y cells with IBU, NAC or AAP for 30 min before OXA incubation for an additional time of 24 h resulted in a complete inhibition of TNF-α production and 56–79% inhibition of IL-6 production (Table 1). Inhibition of IL-6 and IL-1β release was more important with NAC and AAP than with IBU. In addition, PGE2 release in the supernatant of OXA (200 µM)-treated macrophages RAW 264.7 was nearly totally inhibited with NAC, or AAP, or IBU (Table 1).

### Brain tissue analysis in oxaliplatin-treated C57BL/6 mice

**Figure 6:**
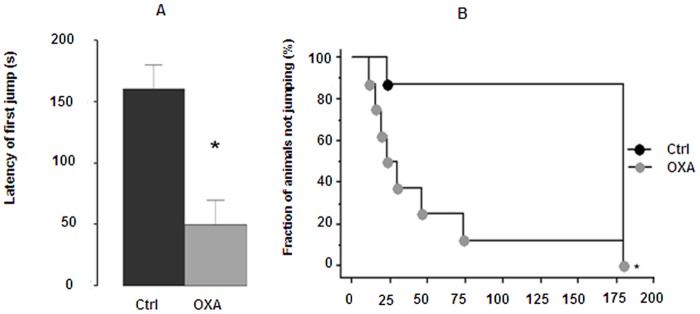
Behavioral assessment of cold hyperalgesia in oxaliplatin-treated C57BL/6 mice using the cold plate test. Mice were repeatedly injected i.p. with 7 mg/kg oxaliplatin (OXA) at days 1, 2, 5 and 6 (28 mg/kg cumulated dose). The latency of first jump was used to evaluate the painful response at 2°C (A), values represent mean ± SEM, * p<0.01. A cut off was set at 3 min in order to avoid tissue damages (B); results are presented as Kaplan-Meier survival curve, * p<0.001.

**Figure 7:**
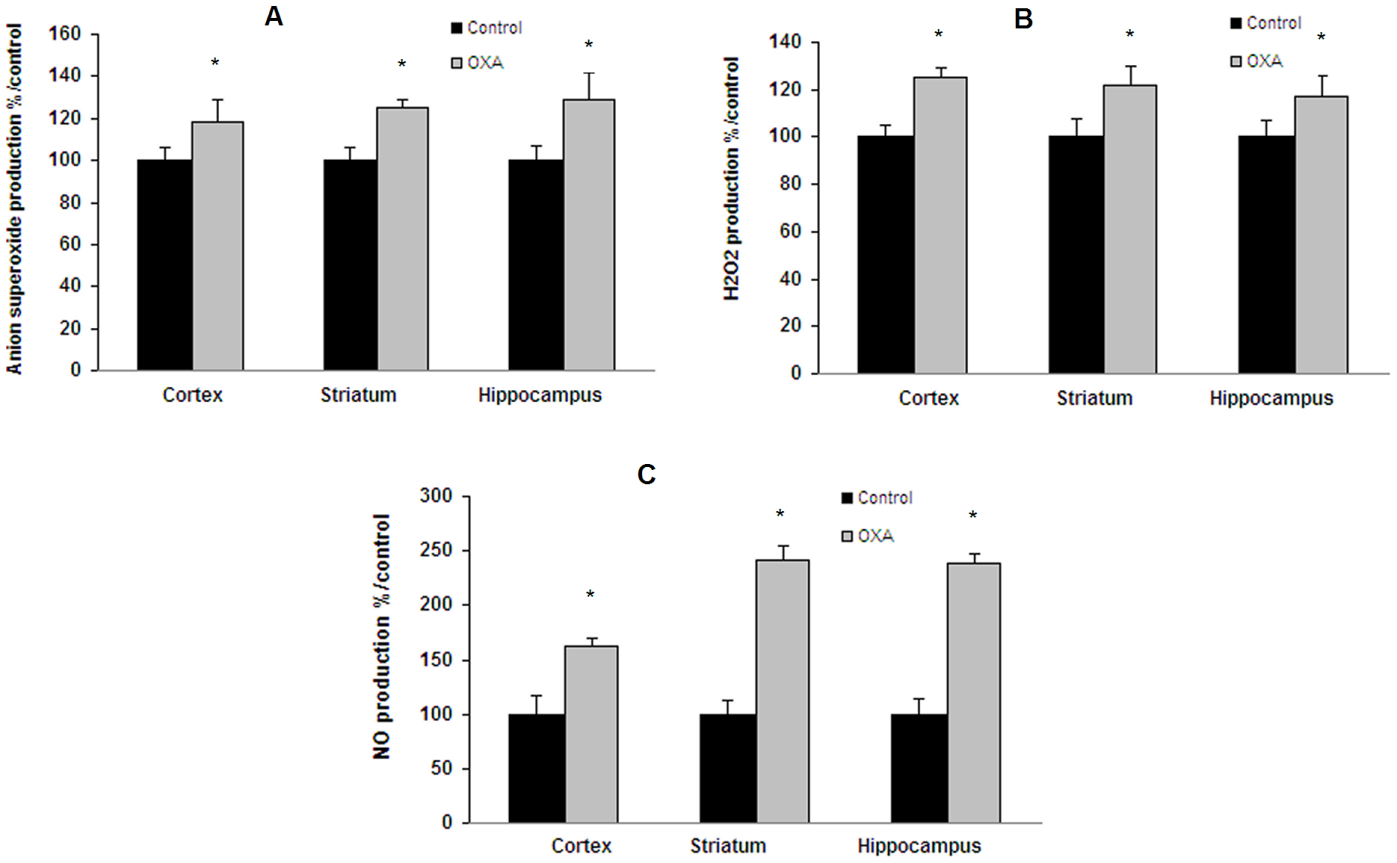
Oxidative stress and mitochondrial activity in oxaliplatin-treated C57BL/6 mice. Mice were repeatedly injected i.p. with 7 mg/kg oxaliplatin (OXA) at days 1, 2, 5 and 6 (28 mg/kg cumulated dose; n = 10). Oxidative stress was evaluated by ROS production using dihydroethidium (A) and DCF-DA (B) tests and NO content (C) by the Griess reaction. Values are the mean ± S.E.M. expressed as percentage of the control (n = 8). *: statistically different (p<0.05) from the mean values in control mice.

**Figure 8:**
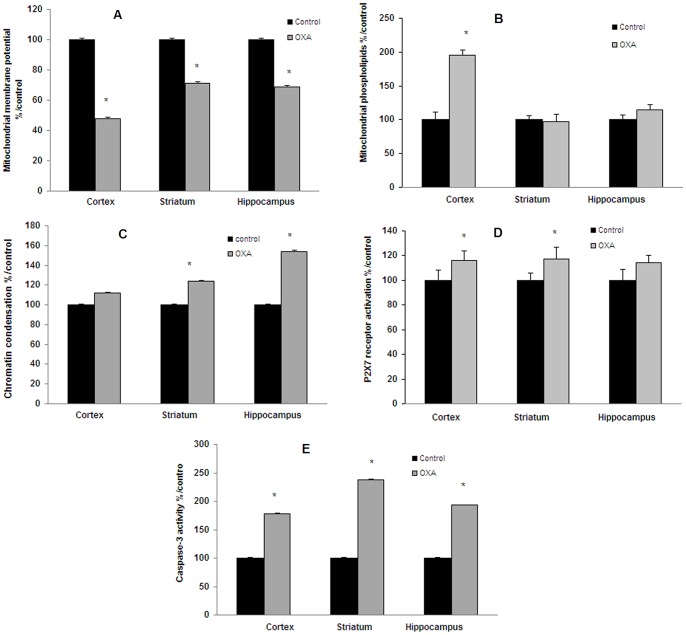
Chromatin condensation, P2X7 receptor activation and caspase-3 activity in oxaliplatin-treated C57BL/6 mice. Mice were repeatedly injected i.p. with 7 mg/kg oxaliplatin (OXA) at days 1, 2, 5 and 6 (28 mg/kg cumulated dose; n = 10). Mitochondrial activity was evaluated by determining mitochondrial membrane potential (A) using JC-1 test and mitochondrial levels of negatively charged phospholipids (B) using nonyl acridine orange test. Chromatin condensation (C) was evaluated using Hoechst 33342 test and P2X7 receptor activation (D) using YOPRO-1 test. The apoTarget^TM^ Caspase-3 Protease assay was used for the *in vitro* determination of caspase-3 proteolytic activity (E) in lysates of brain mitochondrial homogenates as described by the manufacturer's instructions. Values are the mean ± S.E.M. expressed as percentage of the control (n = 8). *: statistically different (p<0.05) from the mean values in control mice.

In the cold plate test (2±0.3°C), repeated injections of oxaliplatin (28 mg/kg as cumulated dose) resulted in a significant decrease in the latency of first jump as compared with control mice (49.9±20.0 *vs* 160.4±19.6 sec; p<0.001) (Fig. 6A). These results showed a significant cold hyperalgesia (Fig. 6B). Treatment of male C57BL/6 mice also caused a significant increase in ROS and NO production both in cortex, striatum and hippocampus (Fig. 7A, 7B, 7C). After JC-1 test, a decrease in green fluorescence (57% in cortex and ≈40% in striatum and hippocampus) was observed, indicating mitochondrial depolarization (Fig. 8A). A small decrease in red fluorescence was also measured in the three tested brain areas (11%, 18% and 22%, respectively). These changes led to a decrease in mitochondrial membrane potential (green/red fluorescence intensity ratio) in the tested brain areas (52% in the cortex and ≈40% in striatum and hippocampus) (Fig. 8A). Mitochondrial levels of negatively charged phospholipids were only increased in brain cortex (95%) (Fig. 8B). Chromatin condensation was increased by 24% and 52% in striatum and hippocampus, respectively (Fig. 8C). P2X7 receptor activation exhibited a tendency to increase (≈15%) with OXA treatment (Fig. 8D) and caspase-3 activity was significantly increased by 78%, 138% and 193% (Fig. 8E) in the three tested brain areas (cortex, striatum and hippocampus, respectively).

## Discussion

Our experiments show that OXA, a widely used chemotherapeutic drug which unfortunately induces neuropathic pain, has direct neurotoxic effects both *in vitro* and *in vivo*. *In vitro*, neuronal cell viability was slightly decreased (10% up to 20%) with OXA concentrations between 5 and 25 µM and to a greater extent (54% up to 70%) at the highest concentrations (between 50 and 200 µM). The impairment in membrane integrity after an incubation time of 24 hours of SH-SY5Y cells leads to disruption of intracellular redox potential. Overall, this decrease in cell viability appeared at the lowest doses and became more important with the highest doses of OXA, particularly from 25 µM.

The OXA concentration range in this study (2–200 µM) was similar to that found in the plasma of treated patients, especially for the six lowest doses (i.e. 2 up to 50 µM). Effectively, plasma Cmax value reached 12 µM after a 130 mg/m^2^ OXA infusion [68], [69]. According to other authors, plasma concentrations were close to 4 µM, 2 hours after 85 mg/m^2^ infusion [70]. In cell lines, IC50 of cell proliferation was 11.6±4.2 µM [71]. However, concentrations higher than 50 µM may be present in tissues, because OXA can accumulate in areas outside the circulation which can lead to an intensive uptake of the drug into body tissues [72]. The dose range was also adjusted to reproduce the characteristics of allodynia to cold as in OXA-treated patients.

The increase in ROS, NO and MDA production was suggestive of oxidative stress and destabilization of membrane phospholipids. Maintaining mitochondrial integrity is essential for cell survival. We have also shown that the mitochondrial membrane potential and metabolism were decreased and were indicative of an inhibition of mitochondrial energization. Therefore, the ability of the mitochondria respiratory chain complexes to maintain steady-state ATP concentration and mitochondrial integrity can be seriously affected by highest OXA concentrations. In contrast, low OXA concentrations failed to show any significant changes, except increased mitochondrial membrane potential and mitochondrial negatively charged phospholipids associated with a slight activation of P2X7 receptor which may be early markers of mitochondrial injury. Various authors [73], [74] have shown that apoptosis is generally linked with a decrease in mitochondrial cardiolipin levels. However, the methods used by these authors allow to determine specifically cardiolipin levels in the inner mitochondrial membrane. The increase we observed in negatively charged phospholipids is indicative of lipid metabolism impairment in mitochondrial function. This result was obtained using the NAO probe which binds to negatively charged phospholipids, mainly cardiolipin associated with a very high association constant (Ka = 2.10^6^M^−1^). The high cardiolipin-NAO association constant may prevent the binding of the dye to other charged phospholipids *in situ,* notably phosphatidylserine and phosphatidylinositol which are also present in the sample but with a much lower association constant (Ka = 7.10^4^M^−1^). [75]. As NAO can pass through the lipid bilayer and bind all of the cardiolipin residues present on the internal surface of the mitochondrial inner membrane [73], we can hypothesize that the global increase in these phospholipids constitutes a deleterious effect resulting from their externalization with the formation of lipid scaffolds for the anchoring of proapoptotic molecules [76].

The P2X7 receptor plays a pivotal role in chronic pain states [77] by initiating or maintaining pathological changes as a result of inflammatory or neuropathic insults [24]. The important increase in P2X7 receptor activation (300% up to 400% apoptosis) observed with the highest concentrations of OXA (50–200 µM), demonstrates a strong membrane disruption which is associated with necrotic cell death (54 up to 70% as seen in the neutral red test). At the lowest OXA concentrations (2–25 µM), corresponding to the therapeutic range, apoptosis was increased by 55% up to 200% and necrosis by only 10% up to 20% (as seen in the neutral red test). The change in the permeability of the plasma membrane would indicate the opening of the P2X7R channel pore which may be associated with P2X7 receptor activation resulting in apoptosis. Such a link between P2X7 receptor activation and apoptosis has been recently shown in neurodegenerative diseases [78]–[79]. In our study, involvement of P2X7 receptor was further confirmed by the inhibitory effect of Brillant Blue G on the activation of this receptor. In contrast, when incubated with the P2X7 agonist 2′,3′-O-(4-benzoyl-benzoyl)-ATP (bzATP) at 200 µM, the SH-SY5Y cell line respond to purinergic stimulation with an increase in intracellular [Ca^2+^] which is mediated by influx of Ca^2+^ *via* P2X7 purinoceptor operated ion channels [21].

The persistent oxidative stress and NO formation in mitochondria could trigger the permeability transition pore opening and release of inter-membrane proteins initiating the apoptotic cascade *via* activation of caspases. Effectively, exposure of SH-SY5Y cells to OXA (50, 100 or 200 µM) led to the activation of caspase-3, which serves as a critical marker for apoptosis. As shown by Donzelli et al. [15], increased caspase-3 activity occurs prior to neuronal cell death. Reduction of Δψ_m_ seems to coincide with massive cell death. Therefore, the accumulation of ROS could be implicated in apoptotic cell death as it was previously demonstrated [80]. Our analysis in SH-SY5Y cells also revealed significant TNF-α and IL-6 release (almost 2.5-fold and 6-fold increase, respectively), while IL-1β release was only slightly increased. These pro-inflammatory cytokines have been shown to induce acute and chronic hyperalgesia and allodynia [81]. Our results suggest that activation of neuronal cells and probably microglial cells under OXA-treatment induces the release of pro-inflammatory cytokines which could be involved in neuropathic pain, with IL-6 and TNF-α as the predominant pro-inflammatory cytokines. Such elevated levels of TNF-α were previously observed with OXA-induced neuropathic pain [82]. TNF-α was also reported to produce mitochondrial-dependent effects on dorsal horn neurons involved in descending pain controls [83].

The majority of the OXA biochemical effects observed in neuronal cells was also found in the macrophage cell line RAW 264.7 with a comparable intensity indicating that macrophages may also participate in OXA-induced oxidative injury. It is known that macrophages infiltrating tumors contribute to oxidative stress in carcinoma cells by producing oxygen radicals [84] and may also be involved in neuropathic or inflammatory pain [38], [85]. Invading macrophages produce and release a large amount of inflammatory mediators which acutely and chronically affect the function of dorsal root ganglion (DRG) by locally stimulating the *en passant* axons in injured nerves. Cyclooxygenase 2 (COX2) was then dramatically increased in invading macrophages in various neuropathic pain models [86]. The role of prostaglandins, formed through the action of both isoforms of the cyclooxygenase (COX) enzyme in the pathophysiology of pain is well documented [87] and COX-2 is up-regulated. In our study, PGE2 levels were significantly higher in OXA-treated macrophages than in control cells. High levels of PGE2 might stimulate nociceptors contributing to the genesis of neuropathic pain by facilitating the synthesis of pain-related molecules including cytokines. The higher TNF-α and IL-6 levels we observed in OXA-treated cells may be relevant with this hypothesis. Pretreatment of neuronal cells with IBU, a nonselective COX-2 inhibitor, which suppressed TNF-α release and reduced IL-6 are also in agreement with this hypothesis and therefore suggest that PGE2 is involved in the release of these cytokines. St-Jacques and Ma [88] have also noticed that facilitating the synthesis of IL-6 in DRG neurons is likely to be a novel mechanism underlying the role of injured nerve-derived PGE2 in the genesis of neuropathic pain. These *in vitro* data suggest that the P2X7/Inflammasome pathway plays an ongoing role in oxaliplatin-induced injury. There is considerable evidence suggesting that P2X7 receptor activation causes caspase-1 activation, which in turn cleaves pro-IL-1β into mature IL-1β, which is then released [89], [90]. Caspase-1, which is also involved in the induction of COX-2 expression and PGE2 production, is a key target to control inflammatory pain [91]. Further exploration of caspase-1/inflammasome should be performed in the future. Nevertheless, because P2X7 cell death receptor and caspase-3 activities are enhanced both in neuronal cells and in macrophages 264.7, we can thus assert that apoptotic cell death can be initiated by two alternative convergent pathways: the extrinsic pathway, which is mediated by cell surface death receptors, and the intrinsic pathway, which is mediated by mitochondria.

A better understanding of the various mechanisms of OXA side effects can contribute to a beneficial therapeutic management. Therefore, we have studied the preventive effects of anti-inflammatory and anti-oxidative drugs with high doses of OXA. The pivotal role of ROS in neuronal injuries has been demonstrated by the beneficial impact of N-acetyl cysteine (NAC) in the treatment of paclitaxel-induced neuropathy [92]. Analgesics like acetaminophen (AAP), and non-steroidal anti-inflammatory drugs (NSAIDs) like ibuprofen (IBU), are also widely used in the treatment of pain and inflammation but they have different side effects. NSAIDs can cause serious adverse drug reactions and IBU was reported to slightly affect mitochondrial function [93]. To date, there is little evidence that such drugs as AAP or IBU have any therapeutic effect. IBU was shown to reverse PGE2 levels in injured nerves and DRG, but the effect on pain behaviour was modest [94]. Our data revealed that AAP, IBU or NAC pre-treatment of OXA-treated cells significantly reduced or blocked all the toxic effects of OXA-treated neuronal SH-SY5Y cells and significantly prevented the increase in TNF-α. NAC and AAP also prevented the increase in IL-6 and IL-1β whereas IBU was less effective. Based on the data observed in this study, each drug could play a beneficial role in preventive OXA-induced apoptosis in the SH-SY5Y cells through inhibition of P2X7 receptor activation by blocking ROS production and caspase-3 activation but also reducing inflammatory response. Overall, NAC has a higher preventive effect in SHSY5Y cells. However, it may be considered that antioxidant therapy could be successful as an effective therapeutic strategy without reducing anti-tumor efficacy, as recently suggested with paclitaxel [92], bortezomib [95] and cisplatin [30]. Nevertheless, this point remains to be investigated with the three tested drugs.

In mice, we have shown that oxaliplatin chronically administered showed significant behavioural nociceptive signs which were consistent with clinical symptoms. OXA caused cold hyperalgesia similar to symptoms reported by patients [96], [97] and to what has been found in previous studies in mice. Effectively, cold hyperalgesia was demonstrated in OXA-treated mice, by Ta et al. [97] at −4.2°C, and Ushio et al. [98] by the acetone test. OXA also induced oxidative stress through ROS and NO production, loss of mitochondrial trans-membrane potential and P2X7 receptor activation resulting in neuronal cell death and apoptosis in the three tested brain areas. While the importance of the interaction of the various mitochondrial functions for our understanding of the pathogenesis of painful neuropathy remains to be elucidated, they have been implicated in nociception [99]. As indicated by Schwartz et al. [100], it is likely that elevated ROS levels lead the redox status of DRG toward oxidation and thus change the signal transduction pathway toward sensitization. Our results in cultured cells suggest that macrophages could participate in the mechanisms of the neuropathic pain we have observed in these mice. It appears that the P2X7 receptor could play a pivotal role in initiating or maintaining pathological changes as a result of inflammatory or neuropathic insults. These receptors are under investigation as potential therapeutic targets for the alleviation of pain [101]. However, genetic and pharmacological evidence in mice and humans converged to indicate that P2X7 receptor pore formation is the main key in chronic pain [102]. Besides, transient receptor potential (TRP) could contribute to the development of thermal hyperalgesia [6]. TRP sensitization was shown mediated by increased intracellular cyclic AMP [103]. A cortical down regulation of potassium channels could also underlie pain chronicity [7].

**Figure 9:**
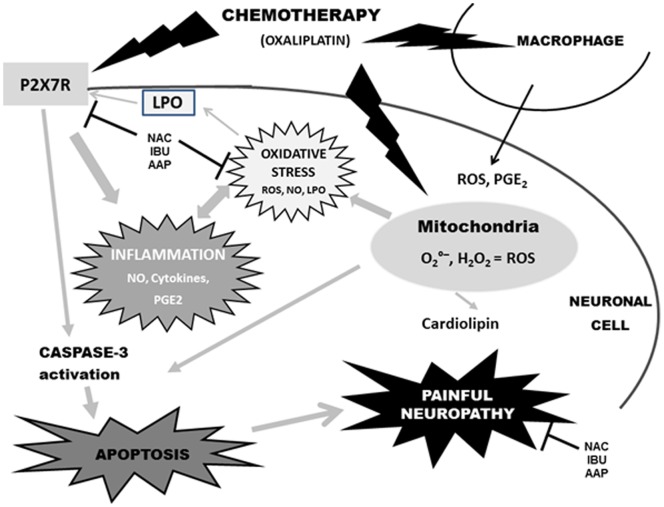
Hypothesis of pathophysiological mechanisms of OXA-induced pain neuropathy. OXA-induced pain neuropathy seems mediated by cell death P2X7 receptor activation and mitotoxicity which induce apoptosis. Symbols: inhibiting pathway; LPO: lipoperoxydation; AAP: acetaminophen; IBU: ibuprofen; NAC: N-acetyl cysteine.

The results presented in our study and recent data of the literature demonstrate that the chronic OXA neurotoxicity at a cellular level may be explained not exclusively by DNA damage but also by the cell activation of molecular pathways which lead the cell to die by apoptosis [15] but also *via* oxidative stress as recently reported [8], [16]. However, to our knowledge, we are the first to report both *in vivo* and *in vitro* the main interaction between mitochondrial dysfunction, P2X7 receptor activation, oxidative stress, inflammation and apoptosis, all together playing a crucial role in OXA-induced neuropathy. In line with this, all our results lead us to hypothesize the pathophysiological pathways of OXA-induced pain neuropathy as shown in Figure 9.

In conclusion, for the first time, our *in vitro* and *in vivo* results provide support for the involvement of an oxidative stress through P2X7 receptor activation and mitochondrial dysfunction in the pathophysiology of OXA-induced neuronal injury and likely to painful neuropathy. Prior oxidative stress/inflammatory response could also be involved in the transition from acute to chronic OXA-induced pain. Mitochondrial impairment as well P2X7 receptor activation could be new potential therapeutic targets for OXA toxicity and probably for the different painful symptoms. Involvement of Macrophages in OXA-induced effects could account for painful neuropathy. COX-2 could be an additional target of OXA cytotoxicity. Drugs which block these targets may have the potential to deliver broad-spectrum analgesia. Although AAP and IBU significantly reduced or blocked all the neurotoxic effects of OXA by reducing oxidative injury and avoiding energy metabolism disturbances, NAC could have a higher beneficial preventive effect to the reduction of painful symptoms which remains to be evaluated in mice.
